# Metagenomic next-generation sequencing reveals co-infection with Legionella pneumophila and Fusobacterium necrophorum in a patient with severe pneumonia: a case report

**DOI:** 10.1186/s12890-024-03097-4

**Published:** 2024-06-12

**Authors:** Yunqi Pan, Yi Xing, Yanan Lai, Huixing Dong, Huiming Sheng, Weihong Xu

**Affiliations:** 1 Department of Laboratory Medicine, Tongren Hospital, Shanghai Jiao Tong University School of Medicine, Shanghai, 200336 China; 2 Department of Hospital Infection Management, Tongren Hospital, Shanghai Jiao Tong University School of Medicine, Shanghai, 200336 China; 3 Department of Respiratory and Critical Care Medicine, Tongren Hospital, Shanghai Jiao Tong University School of Medicine, Shanghai, 200336 China

**Keywords:** *Legionella pneumophila*, *Fusobacterium necrophorum*, Coinfection, Sepsis, Metagenomic next-generation sequencing

## Abstract

**Background:**

Legionella pneumonia is one of the most severe types of atypical pneumonia, impairing multiple organ systems, posing a threat to life. Diagnosing Legionella pneumonia is challenging due to difficulties in culturing the bacteria and limitations in immunoassay sensitivity and specificity.

**Case presentation:**

This paper reports a rare case of sepsis caused by combined infection with *Legionella pneumophila* and *Fusobacterium necrophorum*, leading to respiratory failure, acute kidney injury, acute liver injury, myocardial damage, and electrolyte disorders. In addition, we systematically reviewed literature on patients with combined Legionella infections, analyzing their clinical features, laboratory results and diagnosis.

**Conclusions:**

For pathogens that require prolonged incubation periods and are less sensitive to conventional culturing methods, metagenomic next-generation sequencing (mNGS) can be a powerful supplement to pathogen screening and plays a significant role in the auxiliary diagnosis of complex infectious diseases.

**Supplementary Information:**

The online version contains supplementary material available at 10.1186/s12890-024-03097-4.

## Background

*Legionella pneumophila* is a community-acquired, opportunistic, and pathogenic nontypical microorganism [1, 2]. Most cases are infected by inhaling aerosols produced by shower or cooling water systems, and age, underlying diseases, alcoholism, smoking, or immune suppression are common risk factors [3]. Culture of lower respiratory tract specimens is still the gold standard for detecting Legionnaires’ disease. However, this method requires several days and complex culture media to promote pathogen growth [4], resulting in a lower positivity rate of culture.

*Legionella pneumophila* is the pathogen of severe Legionnaire’s disease pneumonia, and the outcome of the infection mostly depends on bacterial virulence factors and host immunity. Elderly people, immunocompromised patients, and patients with chronic lung diseases are more likely to progress to severe pneumonia. Some cases also cause various extrapulmonary manifestations, known as disseminated Legionnaire’s disease, and severe cases can lead to multiorgan dysfunction and even life-threatening conditions [5]. The mortality rate of Legionella pneumonia increases from 10% in the general population to 50% in the intensive care unit [6].

In recent years, there have been multiple reports of Legionella pneumonia combined with other pathogen infections, including viral, bacterial, and fungal coinfections [7–15], but the symptoms are atypical and easily confused with other pathogen infections. mNGS is widely used in the diagnosis of rare pathogens, providing important diagnostic and therapeutic clues for difficult and critically ill patients.

Here, we report a case of Legionella pneumonia and suspected combined with Lemierre’s syndrome (LS). The pathogenic microorganisms were identified as *Legionella pneumophila* and *Fusobacterium necrophorum* by mNGS testing of bronchoalveolar lavage fluid (BALF) and peripheral blood samples. We then reviewed relevant literature on other pathogenic bacterial coinfections with Legionella.

## Case presentation

**Fig. 1:**
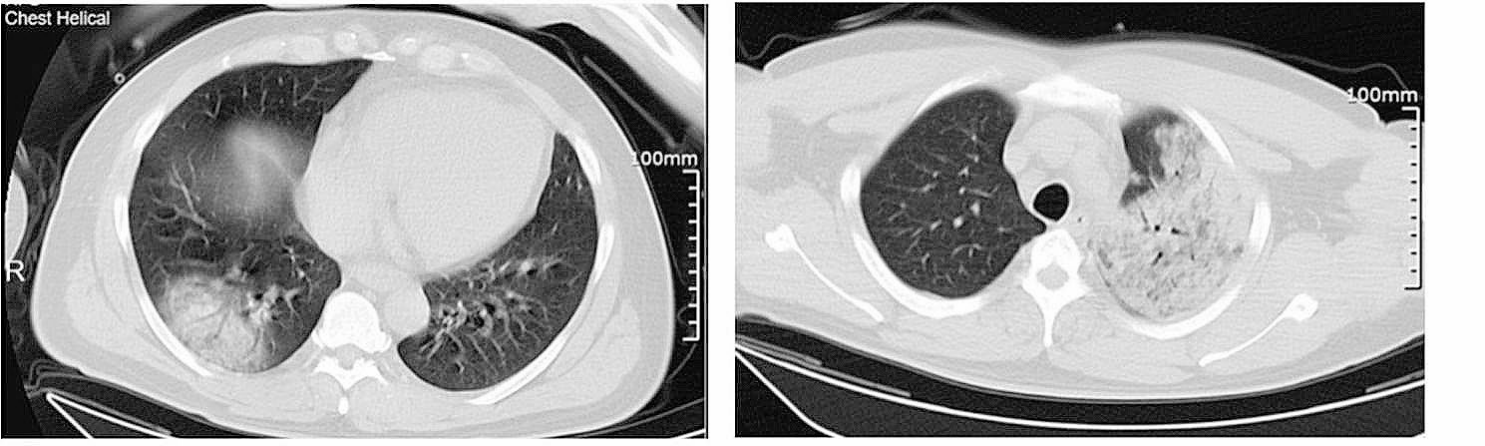
Chest computed tomography scan at admission

A 37-year-old male patient presented to our emergency department on October 31st with symptoms of fever, shortness of breath, and altered consciousness. According to his companion, the patient had developed a fever two days prior, with a maximum temperature of 39.9 °C, occasional coughing, and throat pain, but without significant sputum production. He did not seek medical attention promptly. The patient had a history of hypertension and did not give any history of travel. Physical examination revealed a temperature of 39.2 °C, pulse rate of 120 beats/min, respiratory rate of 32 breaths/min, blood pressure of 151/103 mmHg, coarse breath sounds in both lungs, blue bruises visible on the right side of the abdomen, and no abdominal tenderness. Laboratory testing showed a white blood cell count of 10.69 × 10^9^/L (ref, 3.5–9.5 × 10^9^/L), platelet count of 103 × 10^9^/L (ref, 125–350 × 10^9^/L), C-reactive protein level of 338.99 mg/L (ref, 0–10 mg/L), D-dimer level of 2.34 µg/mL (ref, 0-0.5 mg/L), and procalcitonin level of 5.23 ng/mL (ref, 0–0.5 ng/mL). Chest computed tomography (CT) revealed pneumonia with consolidation (Fig. 1), while head CT and abdominal CT scans showed no obvious abnormalities. The initial diagnosis was severe community-acquired pneumonia.

**Fig. 2:**
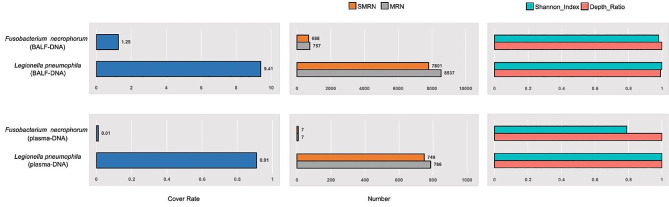
The cover rate, read distribution, Shannon_index and depth ratio of *Legionella pneumophila* and *Fusobacterium necrophorum* in BALF and plasma

Given the patient’s persistent high fever (peaking at 43 °C), symptoms of altered consciousness, and abnormal blood count and chest CT results indicating pneumonia, atypical pneumonia was highly suspected, potentially caused by Legionella or Mycoplasma pneumoniae. Empirical treatment with moxifloxacin (0.4 g daily) was initiated. On November 1st, the patient’s condition deteriorated significantly. Laboratory tests showed arterial oxygen tension of 8.8 KPa (ref, 4.67-6 KPa), blood oxygen saturation of 94.3% (ref, 95–98%), procalcitonin of 6.99 ng/ml (ref, 0-0.5 ng/ml), creatinine at 163.5 umol/L (ref, 57–111 umol/L), aspartate aminotransferase at 119 U/L (ref, 15–40 U/L), creatine kinase at 1728 U/L (ref, 50–310 U/L), lipase at 411 U/L (ref, 13–63 U/L), D-dimer at 9.22 mg/L (ref, 0-0.5 mg/L), calcium at 1.84 mmol/L (ref, 2.11–2.52 mmol /L), phosphorus at 3.08 mmol/L (ref, 0.85–1.51 mmol /L), magnesium at 1.25 mmol/L (ref, 0.75–1.02 mmol/L), and lactate level exceeding 12 mmol/L (ref, 0.9–1.7 mmol/L). A concomitant bloodstream infection, septic shock, and central nervous system infection was suspected. Urgent measures undertaken included colloid albumin supplementation, intravenous immunoglobulin support, and aggressive fluid resuscitation. Due to the severity of the patient’s condition and the possibility of multiple system infection, a combination of doxycycline (0.1 g, 12 h) and meropenem (1.0 g, 8 h) was also administered. The sputum culture showed Streptococcus salivarius and Neisseria species. Blood culture was negative. To ascertain the pathogenic microorganism, bronchoscopy and sputum lavage were performed, and the BALF (Bronchoalveolar Lavage Fluid) and blood samples were sent for mNGS using the PMSeq2000 platform. The next day, mNGS results showed 7801 sequence reads of *Legionella pneumophila* and 688 sequence reads of *Fusobacterium necrophorum* in BALF. Additionally, common respiratory microflora were detected. Unbiased mNGS of the plasma identified 749 sequence reads corresponding to *Legionella pneumophila* and 7 sequence reads of *Fusobacterium necrophorum* were identified, with no fungi, viruses, or specific pathogens detected. The read distribution, cover rate and depth ratio of *Legionella pneumophila* and *Fusobacterium necrophorum* are shown in Fig. 2 .

On November 1st, at 9:40, the patient’s blood oxygen saturation plummeted to 74%, manifesting cyanosis of the lips and respiratory failure. Immediate endotracheal intubation was performed. Despite intensive interventions including chest compressions, inotropic medication, and mechanical ventilation, the patient’s condition rapidly worsened, with subsequent acute kidney and liver failure, cardiac injury, electrolyte imbalances, and eventually, a flatline on the electrocardiogram at 20:30, indicating clinical death.

## Discussion

Legionella is widely distributed in artificial and natural water sources, moist soil, and hospital hot water systems. Legionella can be classified into 58 species and has more than 70 different serogroups. Legionella pneumonia is a multisystem disease with a high mortality rate. The incidence may exhibit an increased prevalence among geriatric populations, patients with comorbidities, tobacco smokers, hospital inpatients, and those with delayed diagnoses and treatment [3, 16]. Chest CT often shows patchy or interstitial infiltrates in the lungs, which can progress to nodular consolidation. After being infected with Legionella, patients may rapidly progress to severe illness and experience mortality due to tension pneumothorax, shock, respiratory failure, or multiple organ dysfunction syndrome [3]. In this case study, the patient presented with fever and consciousness disorders, and CT showed consolidative pneumonia in the lungs. However, a definite diagnosis could not be made based on clinical manifestations alone. Therefore, it is crucial to shorten the time to identify the microorganism for timely diagnosis and appropriate treatment.

Legionella pneumonia is caused by Legionella species, a strict gram-negative bacterium with specific growth requirements. The conventional diagnostic techniques for Legionella pneumonia involve lower respiratory tract culture, urine antigen detection, and quantitative PCR detection of respiratory, serum, or urine samples. Although lower respiratory tract culture is regarded as the reference standard, its sensitivity is restricted [17]. Metagenomic sequencing can significantly improve the early diagnosis of infectious diseases. In this case study, we identified both *Legionella pneumophila* and *Fusobacterium necrophorum* in both plasma and BALF using metagenomic sequencing, providing a basis for pathogen diagnosis.

Considering our coinfection case, Lemierre’s syndrome, also known as postthroat sepsis, is a rare but potentially deadly infectious disease caused primarily by *Fusobacterium necrophorum* [18]. This anaerobic, gram-negative bacterium is commonly found in humans and animals as part of the normal flora of the oral, upper respiratory, gastrointestinal, and genitourinary tracts. LS has an estimated annual incidence of 3.6 cases per million people, with a higher rate of 14.4 cases per million in individuals aged 15–24 years [19]. The condition is more common in men, with a male-to-female ratio of 2:1. *Fusobacterium necrophorum* can produce various toxins associated with abscess formation, arteriovenous thrombosis, metastatic abscesses, and disseminated intravascular coagulation [20]. In early stages of LS, patients may show signs of oropharyngeal infection like fever and throat pain, including peritonsillar abscess. They can also display nonspecific symptoms such as systemic stiffness, chills, swollen neck lymph nodes, limb weakness, as well as gastrointestinal issues like nausea and vomiting [21]. Due to its rapid progression, LS can lead to respiratory failure, requiring advanced life support measures such as respiratory-assisted ventilation after endotracheal intubation. Early identification and targeted therapy are crucial for improving patient outcomes. While blood cultures have a low positivity rate for *Fusobacterium necrophorum*, they remain a vital diagnostic criterion for LS [22]. In this case study, the patient presented with symptoms of fever and throat pain. Detection of Fusobacterium necrosis in BALF and blood mNGS can be inferred that the patient may have complications with LS. However, due to the urgent nature of the patient’s condition, confirmatory imaging for internal jugular vein thrombosis was unavailable.

Regarding Legionnaires’ disease, a previous study found that *Legionella pneumophila* was detected in both BALF and blood samples using mNGS [23]. Therefore, when conventional detection methods fail to identify the pathogen, mNGS should be considered to guide appropriate treatment adjustments [24]. mNGS has been shown to have higher sensitivity than culture methods, especially in blood, BALF and sputum samples [25]. However, the positive mNGS group had a longer hospital stay and a higher 28-day mortality rate, indicating that a positive nucleic acid sequence test may be a potential risk factor for poor prognosis in adult patients. Thus, mNGS should be used more widely for early pathogen detection and diagnosis [26].

The reported cases of coinfection with *Legionella pneumophila* and *Fusobacterium necrophorum* are extremely rare. In fact, coinfection with other pathogens in Legionnaires’ disease is also uncommon, with most cases involving viral coinfection. Here, we review several case series and reports, including seven cases of *Legionella pneumophila* combined with other pathogen infections. Table 1 summarizes their basic features, diagnostic parameters, and prognostic factors. In these cases, Legionella diagnosis tools included PCR-based techniques, urinary antigen tests, and metagenomic sequencing. The studies emphasized the importance but difficulty of diagnosing the pathogen. In most cases, the mortality rate is relatively high when Legionella pneumonia is complicated by other pathogens, and only one patient in the above cases recovered from coinfection. Therefore, early and accurate pathogen diagnosis is extremely important. For patients with severe community-acquired pneumonia, particularly those with multilobar lung lesions and bilateral pleural effusions indicated by imaging, Legionella infection should be considered if initial empirical treatment is ineffective.

**Table 1:** Clinical Characteristics and Diagnosis Parameters of Cases

No.	Gender	Age	Country	Coinfection with pathogens	Diagnostic Tool of Legionnaires’ Disease	Outcomes	Ref.
1	F	> 65	England	SARS-CoV-2	Urine Ag	Expired	Chalker, V.J., et al. [8]
2	F	> 80	England	SARS-CoV-2	Urine Ag	Expired	Chalker, V.J., et al.^9^
3	M	80	Japan	SARS-CoV-2	Urine Ag	Expired	Arashiro, T., et al. [9]
4	M	59	Italy	Klebsiella pneumoniae	real-time PCR	Expired	Scaturro, M., et al. [10]
5	F	43	Portugal	Saprochaete clavata	PCR	Expired	Caldas, J.P., et al. [12]
6	F	40	China	Pneumocystis jirovecii	mNGS	Expired	Oggioni, C., et al. [14]
7	F	80	Caucasian	Streptococcus pneumonia	Urine Ag	Discharge after 1 week	Beg, M., H. Arif, and T. Walsh [15]

## Conclusion

This article discusses the challenges in diagnosing Legionella pneumonia, which can cause severe pneumonia, impair multiple organ systems, and even threaten life. This article reports a rare case of sepsis caused by a combined infection of *Legionella pneumophila* and *Fusobacterium necrophorum*, leading to various complications. The article also reviews the clinical features, laboratory results, and treatment strategies of patients with Legionella combined infection. The article highlights the importance of accurate pathogen detection for effective diagnosis and treatment and suggests that mNGS can be a useful supplement to pathogen screening.

### Electronic supplementary material

Below is the link to the electronic supplementary material.

Supplementary Material 1

## Data Availability

Sequence data that support the findings of this study have been deposited in the NCBI with the primary accession code SRR27909000,SRR27909016,SRR27909063,SRR27909071. Data is provided within the manuscript or supplementary information files.

## Abbreviations

mNGS: metagenomic next-generation sequencing
LS: Lemierre’s syndrome
BALF: bronchoalveolar lavage fluid
CT: Chest computed tomography
